# Study of acoustic and aerodynamic performance of reactive silencer with different configurations: Theoretical, modeling and experimental

**DOI:** 10.1016/j.heliyon.2023.e20058

**Published:** 2023-09-14

**Authors:** Motaleb Rahimi Jokandan, Ali safari variani, Saeid Ahmadi

**Affiliations:** a Occupational Health Engineering, School of Health, Qazvin University of Medical Sciences, Qazvin, Iran; b Department of Occupational Health and Safety, School of Public Health, Qazvin University of Medical Sciences, Qazvin, Iran; c Occupational Health Engineering, Fuman Health and Treatment Network, Guilan University of Medical Sciences, Rasht, Iran

**Keywords:** Extended tube, Baffle, Transmission loss, Insertion loss, Pressure loss, COMSOL

## Abstract

Periodic sound reduction at some frequencies and high pressure loss could be considered as the disadvantages of simple expansion silencers. Generally, reactive elements are applied in silencers to optimize their acoustic and aerodynamic performance. In this paper, we were going to investigate the combined and independent effects of reactive elements, baffle and extended tubes at the inlet and outlet of simple expansion silencer, on the acoustic performance and pressure loss of simple expansion silencer. Firstly, simple expansion silencer dimensions were determined based on theoretical calculations to attenuate the noise by 10 dB. Secondly, the simulation was performed using COMSOL acoustical module software based on finite element method to predict silencer sound transmission loss and pressure loss, respectively, before and after adding reactive elements. Then, according to ISO7235, ISO3744 and ISO3746 standards noise insertion loss of various silencers under study was measured in a free field and in the following that silencers pressure loss evaluated. Finally, the predicted results with the software were compared with the experimental ones. The addition of the extended tubes increased the transmission loss and insertion loss at low and medium frequencies and mitigated the pressure loss of the silencer compared to the simple expansion silencer. The baffle increased the transmission loss curve in the medium frequencies compared to the simple expansion silencer. The combination of baffle and extended tube elements caused an extraordinary increase in insertion loss and transmission loss at the medium frequencies and a decrease in pressure loss. It could be concluded from the present study that using extended tube might be probably the best choice to reduce pressure loss and increase the acoustic performance of simple expansion silencer at low and medium frequencies and what's more is that the best acoustic performance in medium frequencies can be achieved by using a combination of baffle and extended tube.

## 1 Introduction

Noise pollution means unwanted sound, which is considered annoying by most people. Noise pollution not only endangers the lives of most people, but is also one of the important factors of environmental pollution. This affects a person not only physically, but also mentally. For these reasons, noise reduction is very important in societies, and preventing noise is a growing concern in industry and the environment [1].

Noise attenuation in sound generating sources can be categorized as active and passive methods. Passive noise control methods are used more than active ones due to lower costs, small geometries and compatibility with the noise generating device in harsh environmental conditions. Among the types of passive noise control methods, we can mention acoustic silencers, noise barriers and, noise absorbing panels [2]. In the meantime, silencers or mufflers are used to reduce the noise propagated from numerous sources such as internal combustion engines, fans, compressors, turbines, air conditioning systems, blowers and so on [3]. There are various types of silencers which includes reactive, absorptive and hybrid ones. Reactive silencers, which work based on the principle of impedance mismatch, reflect sound waves toward the noise source by suddenly expanding or changing the cross section area [4]. The noise reduction mechanism in absorptive silencers is based on the absorption of sound wave energy that propagates along the tube and converting it into heat through sound absorption materials. Hybrid silencers are constituted from both absorbing and reactive parts to improve the performance [5]. Designing a silencer is a complex process that affect on the acoustic and aerodynamic performance of noise generating devices, so it should be designed in a good manner. The important criteria in the design of the expansion silencer include minimum sound reduction in the desired frequency range, pressure loss, geometry, maximum allowable dimensions, strength and economic aspects [6].

Basically, the acoustic performance of silencers is characterized by parameters such as insertion loss (IL), transmission loss (TL), noise reduction rate (NR). Additionally, aerodynamic performance, commonly known as pressure loss, is measured by the total air flow pressure. The most common parameter used to evaluate the acoustic properties of a silencer is the transmission loss (TL) [7]. The transmission loss can be predicted using the dimensions and physical parameters of the silencer. There are different methods such as analytical and numerical solution methods to evaluate sound transmission loss and pressure loss of silencers [8].

Using analytical methods to evaluate the performance of silencers with complex internal geometry is very exhausting and time-consuming. Numerical solution methods such as finite element and boundary element methods are suitable for predicting the performance of reactive and absorptive silencers with different geometries [9,10]. Middelburg et al. investigated the acoustic and dynamic performance using the computational fluid dynamic method [11]. Geometric optimizations have been performed in various studies [12]. Antebas et al. also investigated the sound propagation in the perforated absorptive silencer using the finite element model [13]. Various software packages for predicting transmission loss and pressure loss such as Abaqus, MATLAB, Fluent, Ansys and, etc. Are available on the market. COMSOL multi-physics software is a complete set of scientific or computer modeling and simulation that is based on the finite element method. This software allows users to enter or draw a desired geometry, applying boundary conditions, select the physical parameters and in the following that solve the dominant equations in one, two or three dimensions [14].

**Fig. 1:**
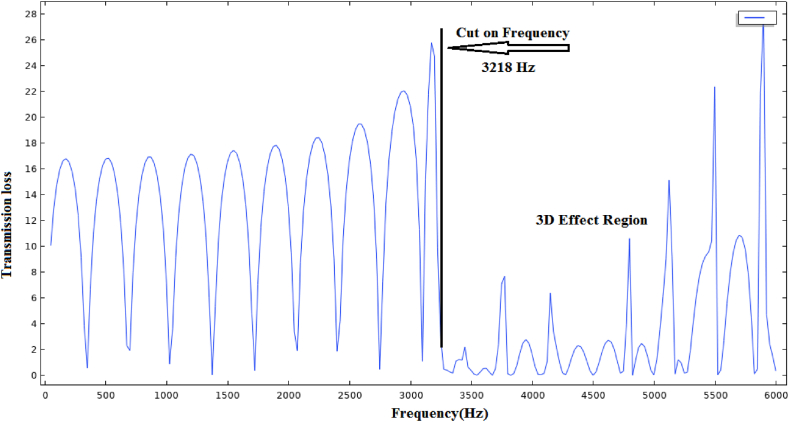
Simple expansion silencer transmission loss curve.

Many researchers have worked to increase the transmission loss as the first goal in the silencer and reduce the pressure loss as the second goal [15]. Assessing the transmission loss curve of the simple expansion silencer (Fig. 1) shows that the amount of transmission loss is the highest in some frequencies and the lowest in some frequencies. Therefore, They could be solely used to reduce the sound in certain frequencies [16]. This periodic fluctuation behavior, like the transmission loss curve of simple expansion chambers, reduces their overall performance. In addition, it should be mentioned that these types of silencers do not have optimal acoustic performance at frequencies higher than the first cut-off frequency.

**Fig. 2:**
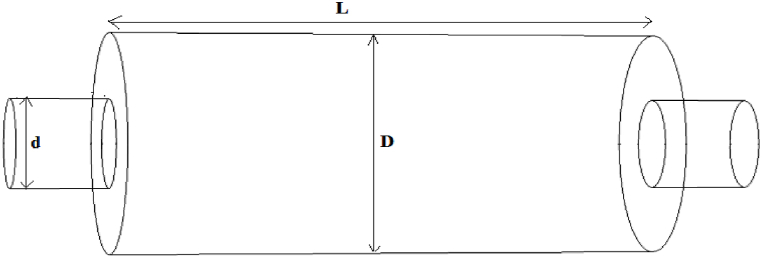
Simple expansion silencer.

One of the ways to increase the transmission loss of simple expansion silencers (Fig. 2) is to increase the cross-sectional area of the expansion chamber compared to the cross-sectional area of the inlet tube. This proportion is in reverse relation with the cut-off frequency, so with increasing expansion chamber diameter cut-off frequency will be shifted toward Lower frequencies and it leads to reduce the performance of the silencer in the frequency range [17]. Additionally, simple expansion chambers have other limitations such as an increase in pressure loss due to a sudden change in cross section area. Pressure loss is one of the important aspects of silencer, which should be considered as a principle during design. Because it can significantly affect its performance [11]. Therefore, to achieve optimal sound reduction, the ratio of the surface of the expansion chamber to the surface of the inlet tube should be considered in the calculations. The noise reduction values by the simple expansion chamber can be obtained from Eq. (1) [2].

(1) $$\text{TL}=10\text{loge}\;\left[1+1/4{\left(m-1/m\right)}^{2}\sin^{2}\left(\text{kl}\right)\right]$$

Where TL represents the value of sound transmission loss, m is the cross-sectional area ratio (D/d), k is the wave number (k = 2π/λ = 2πf/c = ω/c) and L is the length of the expansion chamber in meters. The maximum amount of sound reduction occurs when L equals to 1/4 of the wavelength or an individual number of 1/4 of the wavelength of the sound frequency under investigation. Therefore, by calculating the length of the silencer from Eq. (2) and using Eq. (3), it is possible to calculate the frequencies in which the highest amount of transmission loss will occur [2].

(2) $$L=n\;\frac{\lambda }{4}\;n=1,3,5,7,\ldots \text{.}$$

(3) $$F=\frac{C}{\lambda }$$

in these equations, L is the length of the silencer, λ is the wavelength, F is the frequency, and C is the speed of the sound wave.

Generally, the optimum acoustical performance range of expansion silencer was deemed to be below the first cut-off frequency. The following equation, Eq. (4), is used to determine the first cut-off frequency in simple expansion [18].

(4) $$F_{\text{up}}=1.22C/D$$

In this equation, C represents the speed of sound (m/s) and D is denoted as the diameter of the silencer expansion chamber in (m).

The use of sound-absorbing fibrous materials inside the expansion chamber improves the above mentioned limitations and increases the transmission loss in medium frequencies and frequencies higher than the cut-off frequency. The escape of sound absorbing materials is associated with problems [19]. Selamet et al. investigated the expansion chamber covered with two layers of fibrous absorbers with different flow resistances and thicknesses, the result of which was the improvement of the transmission loss of the silencer in the medium and higher frequencies without a significant decrease in the lower frequencies [20].

Moreover, improvement in the transmission loss of simple expansion silencers could be divided to adding extended tubes at the inlet and outlet of the silencer, baffles, asymmetric inlets and outlets, extended tubes constructed from punched metal sheet and connecting expansion chambers in series [21]. Various studies have been done in different ways [[22], [23], [24], [25]]. AK Gupta investigated the transmission loss in a simple expansion chamber with inlet and outlet extended tubes which had the lengths equal to a quarter of the length of the chamber using COMSOL software based on FEM [14]. Elsayed et al. examined the effect of different baffle sizes on the transmission loss and silencer pressure loss using acoustical module of COMSOL software based on FEM, their result revealed an increase in transmission loss in medium and higher frequencies and a decrease in transmission loss in low frequencies [26]. Utilizing inlet and outlet extended tubes probably is accompanied with resonance in frequencies with lower sound transmission loss, hence it could be led to improve sound transmission loss and pressure loss. The length of extended tubes is an influential parameter in the performance of silencers. It sounds that making longer the length of extended tubes might increase the amount of resonance and eventually improve the performance of silencer. To employ baffle in silencers essential parameters should be considered that includes type, number, size and location.

According to the location and size, the baffles can extremely reflect the sound waves, like the end part of the expansion chamber, toward sound source and subsequently increase the acoustic performance of the silencer. As if adding baffles could be led to an increment in pressure loss, a cavity or hole on the surface of the baffle is usually used to pass the air flow and adjust pressure loss.

However, increasing the length of the extended tube, the type and location of the baffle can improve the performance of expansion silencers, previous work has probably failed to address the acoustic and aerodynamic performance of expansion silencers with different extended inlet and outlet lengths. Moreover, there is some controversy surrounding the acoustic performance and loss pressure of silencers having perforated baffle with the same diameter size as the chamber inlet and outlet tubes. As well as that, it has not been widely understood whether a combination of baffle and extended tubes can optimize silencer performance. Despite this interest, as far as we know no one has studied measuring noise insertion loss in experimental set up with silencers and tubes which had approximately real size. The present paper aims to examine the influence of extended inlet and outlet tube with different sizes, utilizing baffle with a central hole in the middle of chamber and combined aforementioned reactive elements on the acoustic and aerodynamic performance of simple expansion silencers using the computer simulating software COMSOL Multiphysics and the simulation results were verified with experimental ones.

## 2 Materials and methods

### 2.1 Theory

**Fig. 3:**
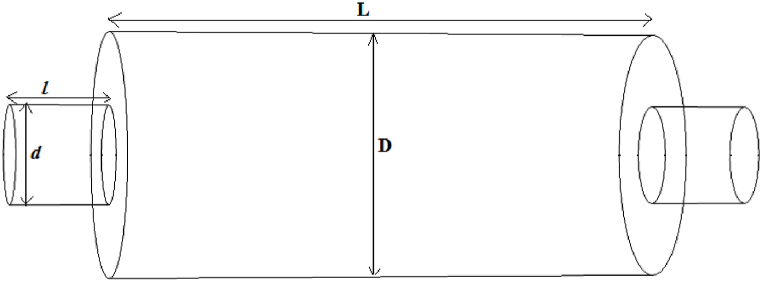
Simple designed expansion silencer.

The length and diameter of a simple expansion were calculated based on Eqs. (1) and (2) for a sound transmission loss of 10 dB (Fig. 3). The length and diameter of the inlet and outlet tubes were considered to be 15 cm. The first cut-off frequency in the simple expansion silencer was calculated by Eq. (4 [2].

**Fig. 4:**
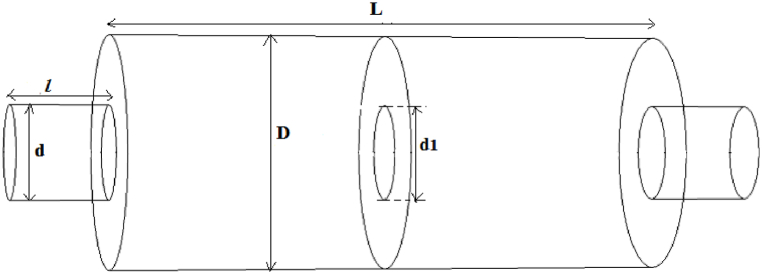
Expantion silencer with baffle.

In order to identify the effect of the baffle on the performance of the simple expansion silencer, a baffle with a central hole of 15 cm in diameter (equal to the diameter of the inlet and outlet (d = d1 = 15 cm)) was formed in the middle of the expansion chamber, so the simple expansion chamber was divided by two simple expansion chambers with the same length. (Fig. 4).

**Fig. 5:**
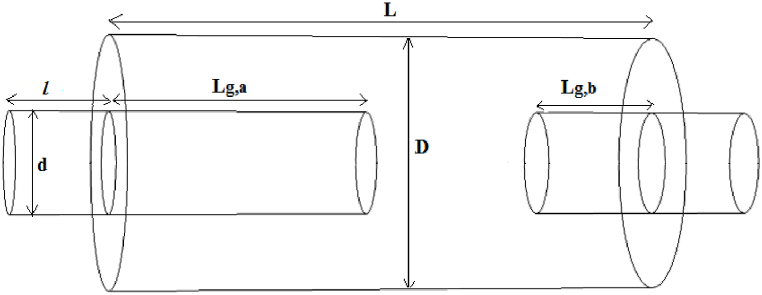
Expansion silencer with extended inlet and outlet tube.

For the silencer with extended inlet and outlet (Fig. 5), the length of the extended inlet and outlet tubes was calculated to be half and a quarter of the length of the chamber, respectively, based on Eqs. (5), (6)) [27].

(5) $$L_{g,a}=\frac{L}{2}-\delta$$

(6) $$L_{g,b}=\frac{L}{4}-\delta$$

in these equations, L_a_ = length of inlet tube, L_b_ = length of outlet tube, L = length of silencer expansion chamber, δ = final correction factor. The final correction factor was calculated from Eq. (7.

(7) $$\frac{\delta }{d}=a_{0}+a_{1}\left(\frac{D}{d}\right)+a_{2}\left(\frac{t_{w}}{d}\right)+a_{3}{\left(\frac{D}{d}\right)}^{2}+a_{4}\left(\frac{D}{d}\frac{t_{w}}{d}\right)+a_{5}{\left(\frac{t_{w}}{d}\right)}^{2}$$

The values of a_0_ = 0.005177, a_1_ = 0.0909, a_2_ = 0.537, a_3_ = −0.008594, a_4_ = 0.02616, a_5_ = −5.425, t_w_ is the thickness of the inlet and outlet tube (0.2 cm), d is the diameter of the inlet and outlet tube and D is the diameter of an expansion silencer.

**Fig. 6:**
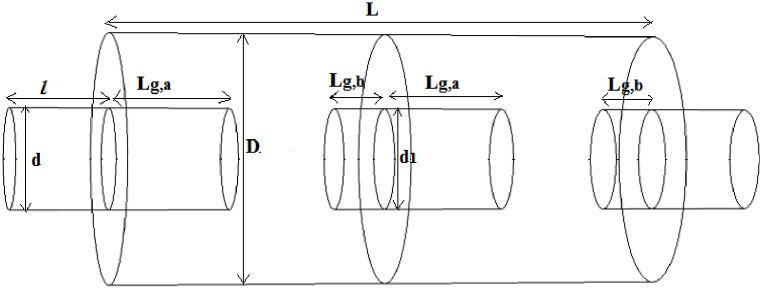
Expansion silencer with baffle and extended inlet and outlet tubes.

Using the central baffle as a resonator to increase the performance of the simple expansion chamber transforms it into two smaller expansion chambers, so the extended tubes at the inlet and outlet of these new smaller chambers will be half and a quarter of the length of the new chambers, respectively. It should be noted that final correction factor for these new smaller chambers were calculated separately based on their new dimensions (Fig. 6).

### 2.2 Modeling

#### 2.2.1 Acoustic section

The geometry of the studied silencers was designed in the pressure acoustics module using COMSOL 5a multiphysics software. The silencer components consist of expansion chamber, inlet and outlet tubes, extended inlet and outlet tubes and, baffle.

##### 2.2.1.1 Governing equations

The acoustic analysis in the frequency domain is governed by Helmholtz equation, equation number 8, which is expressed as follows (Eq. (8)) [[28], [29], [30]].

(8) $$\nabla .\left(-\frac{\nabla p}{\rho }\right)-\frac{\omega^{2}p}{c^{2}\rho }=0$$

where p is the sound pressure, ω is angular frequency, ρ material density and c is the sound speed.

To solve the Helmholtz equation, the sound transmission loss parameter is calculated in different frequencies to show the efficiency of silencer or silencer noise reduction (Eq. (9)).

(9) $$\text{TL}=10\;\log\left(\frac{P_{in}}{P_{out}}\right)$$

$P_{in}$ and $P_{out}$ represents the acoustic power in the inlet and outlet port of the expansion chamber, respectively, and are calculated as follows by Eqs. (10), (11)).

(10) $$P_{in}={ʃ}_{\partial \Omega }\frac{{p_{0}}^{2}}{2\rho c}dA$$

(11) $$P_{out}={ʃ}_{\partial \Omega }\frac{p^{2}}{2\rho c}dA$$

where p_0_ is equal to 1Pa, ρ is the gas density, c is the speed of sound and A is the inlet and outlet port surface area. Sound speed and air density was considered to be 343 m s^−1^, and 1.2 kg m^−3^ in this study respectively.

**Fig. 7:**
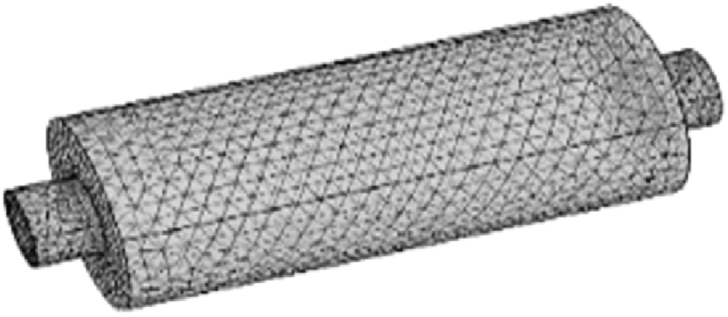
Tetrahedral mesh.

For the meshing of the silencer, a tetrahedral mesh has been selected. The mesh size was assigned to be one fifth of the wavelength with the highest frequency under investigation (λmin/5) (Fig. 7) [31].

##### 2.2.1.2 Surface boundary conditions

1 All solid surfaces that include the outer walls of the expansion chamber, silencer inlet and outlet tubes, baffle and extended tubes were considered as sound hard boundary and soundproof [30].

(12) $$\left(-\frac{\nabla p}{\rho }\right).\;n=0$$

2 A combination of input and output flat waves was considered for the input surface of the silencer (Eq. (13)).

(13) $$\left(-\frac{\nabla p}{\rho }\right).\;n=\frac{i\omega }{\rho c}p-\frac{2i\omega }{\rho c}p_{0}$$

where p_0_ = 1 Pa is the pressure applied at the opening of the inlet tube and i is an imaginary unit. ω is angular frequency, ρ is air density and c is the sound speed.

3 An outgoing flat wave was assigned for the outlet port surface of the silencer (Eq. (14)).

(14) $$\left(-\frac{\nabla p}{\rho }\right).\;n=\frac{i\omega }{\rho c}p$$

#### 2.2.2 Pressure loss section

##### **2.2.2.1** Governing equations

The investigation of the aerodynamic performance (pressure loss) of the silencers was done with a two-dimensional symmetrical model in the form of steady flow. The equations governing this part of the modeling are the Navier-Stokes, Eqs. (15), (16)), which are expressed as follows [[32], [33], [34]].

(15) $$\frac{\partial \rho }{\partial t}+\nabla .\left(\rho u\right)=0$$

(16) $$\rho \frac{\partial u}{\partial t}+\rho u.\nabla u=-\nabla p+\nabla .\left(\mu \left(\nabla u+{\left(\nabla u\right)}^{T}\right)-\frac{2}{3}\mu \left(\nabla .u\right)I\right)+F$$

in these equations u, P, μ and ρ represent the average velocity field (m/s), the average pressure (Pa), the fluid's dynamic viscosity (kg/m.s) and, The fluid's density (kg/m^3^), respectively.

The model applied to solve the Navier-Stokes equations is the k-ε turbulence model such that the kinetic energy of the turbulence k and the amount of wasted energy ε are obtained from solving the transition, which are expressed as follows (Eqs. (17), (18).

(17) $$\rho \left(u.\nabla \right)k=\nabla .\;\left[\left(\mu +\frac{\mu_{T}}{\sigma_{k}}\right)\nabla_{k}\right]+P_{K}-\rho \epsilon$$

(18) $$\rho \left(u.\nabla \right)\epsilon =\nabla .\left[\left(\mu +\frac{\mu_{T}}{\sigma_{\epsilon }}\right)\nabla_{\epsilon }\right]+C_{\epsilon 1}\frac{\epsilon }{k}P_{k}-C_{\epsilon 2}\rho \frac{\epsilon^{2}}{k},\epsilon =ep$$

in these equations, μ_T_ reveals Turbulent viscosity (kg/(m.s)) and is expressed according to Eq. (19. K and ε stands for Turbulent kinetic energy (m^2^/s^2^) and Turbulent dissipation rate (m^2^/s^3^), respectively. The coefficients of these equations are C_€1_ = 1.44, C_€2_ = 1.92, C_μ_ = 0.09, σk = 1 and σ_€_ = 1.3.

(19) $$\mu_{T}={\rho C}_{\mu }\frac{k^{2}}{\varepsilon }$$

P_k_ is turbulence kinetic energy that describes the balance between the production, dissipation, and transport of turbulence kinetic energy in a fluid (Eq. (20)).

(20) $$P_{k}=\mu_{T}\left[\nabla u:\left(\nabla u+{\left(\nabla u\right)}^{T}\right)-\frac{2}{3}{\left(\nabla u\right)}^{2}\right]-\frac{2}{3}\;\rho k\nabla .u$$

in these equation μ, T, u, ρk represent the dynamic viscosity of the fluid, the temperature, the average velocity field.

**Fig. 8:**
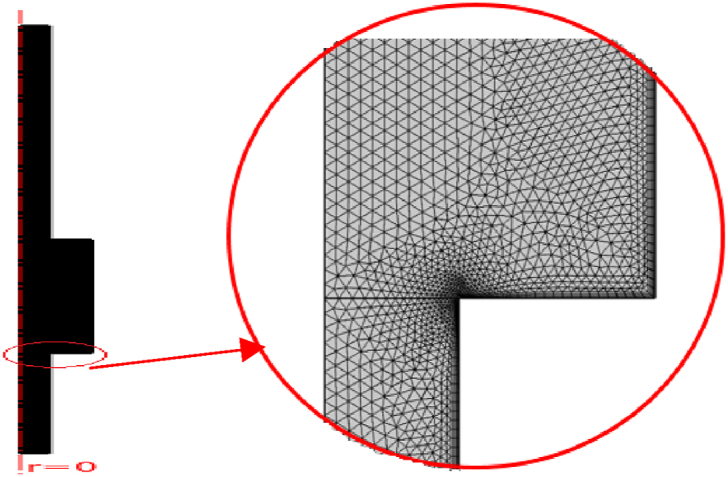
Extra fine tetrahedral mesh.

An extra fine tetrahedral mesh was used for meshing (Fig. 8).

##### 2.2.2.2 Boundary conditions

Air as an ideal gas was used in aerodynamic simulation. Air fluid properties are depicted in Table 1. Boundary conditions include fluid velocity at the inlet and pressure at the outlet and types of walls.

**Table 1:** Fluid characteristics used in the modeling.

Variable	Values	Unit
**Working Fluid**	Air	
**Average Pressure**	101.325	kPa_abs_
**Average Temperature**	293.1	k
**Gas Constant**	287	kJ/kg.K
**Dynamic Viscosity**	1.79.10–5	kg/m.s
**Specific Heat Ratio**	1.40	

### 2.3 Construction

**Fig. 9:**
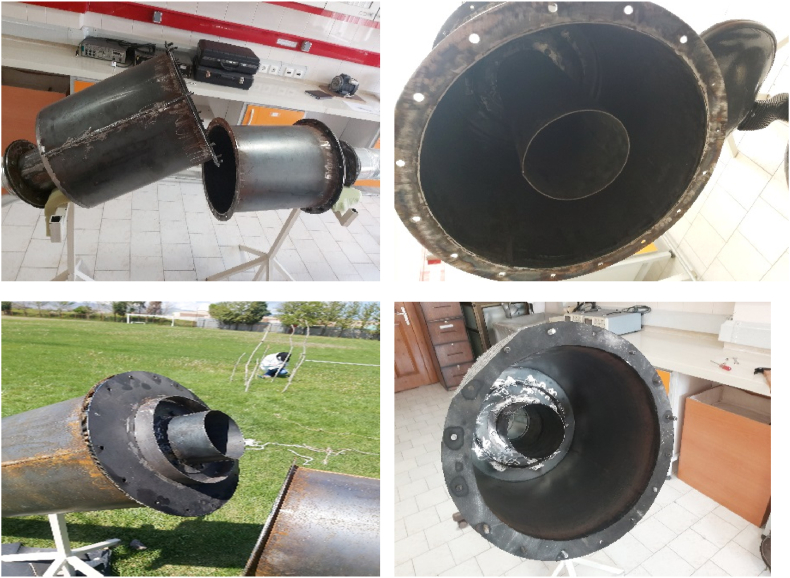
Constructed silencer and reactive elements.

Black iron sheet with a thickness of 0.2 cm and a flange with a thickness of 0.4 cm were used to prepare silencer. Construction and assembly operations were performed by welding and plasma cutting. At first, a simple expansion silencer was fabricated such that the other silencers under study could be assembled by connecting baffles and extended tubes. In other words, a modular acoustic silencer was designed and constructed (Fig. 9)**.**

### 2.4 Experimental method

#### **2.4.1** Insertion loss

**Fig. 10:**
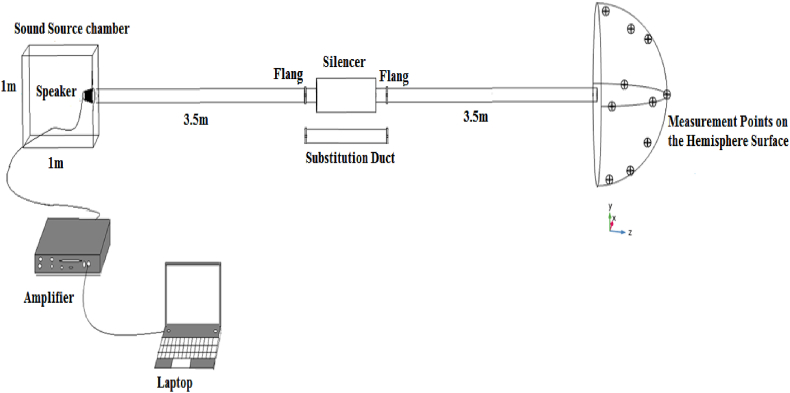
Schematic diagram of experimental measurement of insertion loss of silencers.

Experimental measurement, in accordance with ISO7235, was performed to determine silencers acoustic insertion loss without air flow condition. This particular method has been chosen on account of the fact that insertion loss equals sound transmission loss. An experimental set up and the schematic diagram adopted to evaluate insertion loss was illustrated in Fig. 10. A Sound level meter Casella (UK) type Cell-450, a calibrator type Cell 110/2, speaker as a noise source, Cool Edit noise generation software, sound source chamber, metal ducts and an amplifier constituted acoustic measurement set up. Experiment was carried out in the football field, as a free field, of the Qazvin University of Medical Sciences, Qazvin, Iran.

The sound power level should be measured at two distinct modes at the duct's end, with silencer and while substitution duct replaced with silencer, based on ISO 7235. The difference in the sound power level in the two modes indicates the insertion loss of the silencer. The pink noise was created by Cool Edit noise generation software and amplified by an amplifier before delivering to the loudspeaker as a noise source. It should be mentioned that in order to direct all noise generated by speaker into the inlet duct, speaker was surrounded by an acoustic enclosure. All internal surfaces of acoustic enclosure were covered by sound-absorbing materials. The speaker enclosure was a cubic chamber with a length, width and height of 1 m. It was connected to the inlet duct through a connector. The length of the duct used in the distance between the speaker chamber to the silencer and the silencer to the measurement location (silencer outlet) is equal to 3.5 m [35,36].

Output sound power level of silencer and substitution duct was measured and calculated by ISO3744 and ISO3746 standards in a free field (football field). A microphone array like a hemispherical measurement surface was utilized to measure sound pressure level on 10 different points by a sound level meter. A hygrometer, thermometer and, a PROVA anemometer was used to measure relative humidity, temperature and wind speed, respectively. According to ISO 3744 and based on the fact that the difference between the sound pressure level of the background and sound source was more than 15 dB, the background noise coefficient was considered to be zero and was not applied. Another point was that, referring to the aforementioned standard and taking into account that the measurement was performed in the free field with a reflective plate, the environmental correction factor was also ignored. So, the insertion loss was calculated from Eq. (201 [37,38].

(21) $${IL=Lw}_{2}-{Lw}_{1}$$

Lw_2_ is the sound power level without silencer (with substitution duct), and Lw_1_ is the sound power level with silencer.

#### 2.4.2 Pressure loss

**Fig. 11:**
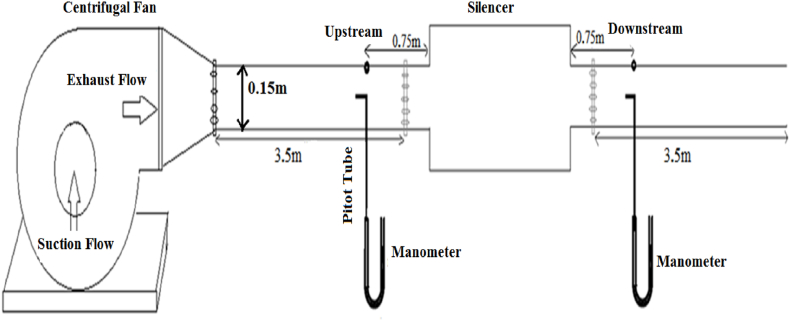
Schematic diagram for experimental measurement of silencer pressure loss.

An experimental set up and the schematic diagram adopted to evaluate the pressure loss of silencers were created, as illustrated in Fig. 11. A backward centrifugal fan (1.1 Kw and 2825 RPM), a pitot tube AFL type 71,805,301, a manometer TESTO type 510, an electric converter, and silencers/substitution ducts comprised loss pressure measurement set up. The adjustment of air flow velocity was conducted by an electric converter to produce various air flow velocities. Mean total air pressure was measured at two places on upstream and downstream of the silencer at various air flow velocities. These places were positioned at a distance 5 times duct's diameter from the silencer. In each side of the silencer, mean total pressure was measured on a plan perpendicular to air flow at 9 different points. The total pressure was calculated from Eq. (212 [39].

(22) $$\Delta \;p=\bar{{\text{pt}}_{1}}–\bar{{\text{pt}}_{2}}$$

in these equations pt_1_ mean total pressure in upstream and pt_2_ mean total pressure is in downstream.

## 3 Results and discussion

### 3.1 Results of theoretical calculations

The results of theoretical calculations for silencers are reported in Table 2. The length and diameter of the simple expansion silencer for reaching a sound transmission loss of 10 dB were 85 and 37.2 Cm, respectively. The first cut off frequency for this silencer was calculated to be 1130 Hz. The final correction factor for extended tubes was 2.8 Cm.

**Table 2:** Theoretical calculation results for dimensions of silencers.

Silencer	Parameter						
	Length(L) *Cm*	Chamber Diameter (D) *Cm*	Inlet Tube Length (l) *Cm*	Inlet tube Diameter (d) *Cm*	Baffle Hole Diameter (d1) *Cm*	Length of Extended Inlet Tube (Lg,a) *Cm*	Length of Extended Outlet Tube (Lg,b) *Cm*
Simple Expansion Silencer	85	37.2	15	15			
Expansion Silencer with Baffle	85	37.2	15	15	15		
Expansion Silencer with Extended Inlet and Outlet Tube	85	37.2	15	15		39.7	18.4
Expansion Silencer with Baffle and Extended Inlet and Outlet Tubes	85	37.2	15	15	15	18.4	7.8

### 3.2 Acoustic performance

**Fig. 12:**
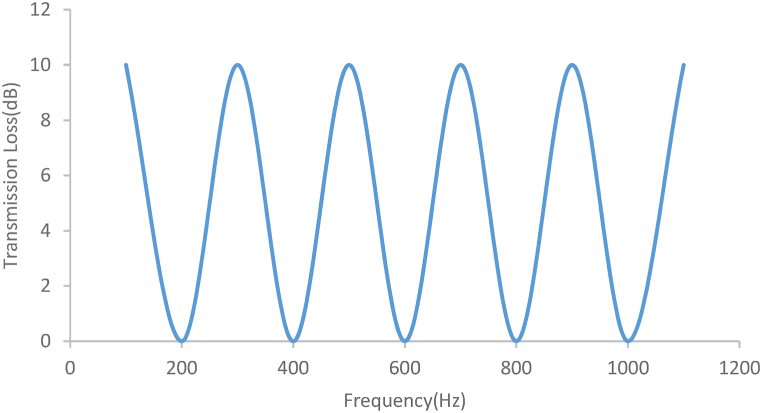
Sound transmission loss curve of simple expansion silencer based on theoretical calculations.

According to the calculations, sound transmission loss curve for the aforementioned dimension of simple expansion silencer was presented in Fig. 12. The highest sound transmission losses were obtained at the frequencies of 100, 300, 500, 700, 900 and 1100 Hz and the lowest ones were obtained at the frequencies of 200, 400, 600, 800 and 1000 Hz.

**Fig. 13:**
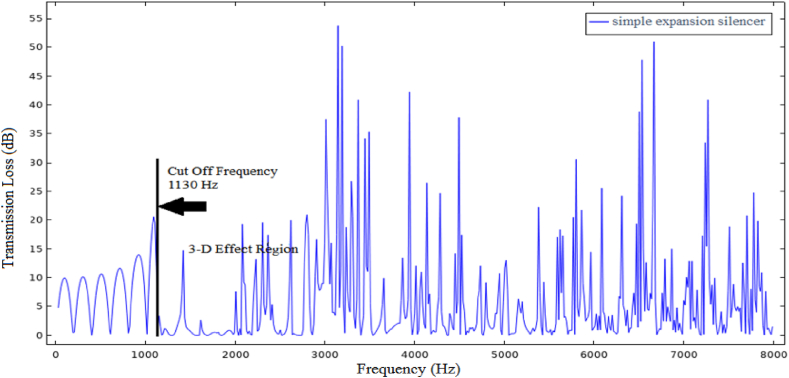
Sound transmission loss curve of simple expansion silencer.

**Fig. 14:**
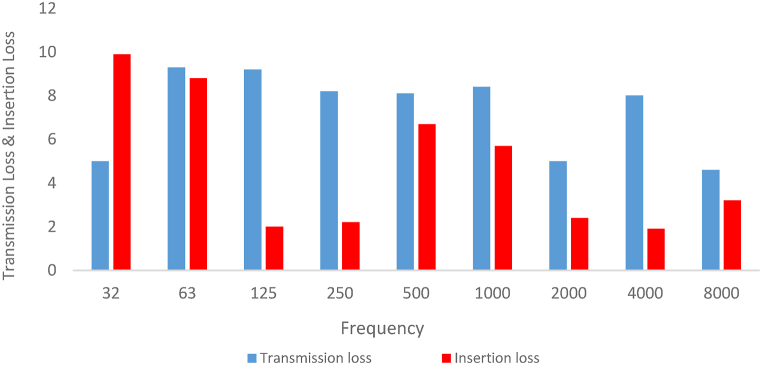
Sound transmission loss and insertion loss of simple expansion silencer in octave band frequencies.

Fig. 13 illustrates the sound transmission loss curve of simple expansion silencer for the frequencies lower than 8000 Hz acquired from finite element method acoustic simulation in COMSOL. As can be seen in Fig. 12, Fig. 13, the sound transmission loss curve of simple expansion silencer shows a sinusoidal behavior and periodic sound reduction of 10 dB in some frequencies. The highest amount of sound transmission loss (10 dB) was observed in the frequencies of 100, 300, 500, 700, 900 and 1100 Hz. On the other hands, the lowest transmission loss (0 dB) was obtained in the frequencies of 200, 400, 600, 800 and 1000 Hz is obtained. Additionally, in order to present further information, the sound transmission loss of simple expansion silencer for octave band frequencies obtained from simulation in COMSOL and Experimental measurement of noise insertion loss is shown in Fig. 14. The average transmission loss in octave band frequencies was 8 dB. Sound transmission loss was a bit different, nearly 2 dB, between octave frequencies and the frequencies with the highest transmission loss. It can nevertheless be argued that highest transmission losses are calculated in specified single frequencies, while in octave frequencies, each octave frequency is a representative or an average of its higher and lower frequencies.

Experimental measurement of noise insertion loss for simple expansion silencer are highlighted. Generally speaking, It can be seen that insertion loss is 9 dB in the frequencies of 32 and 63 Hz, 2–3 dB in the frequencies of 125 and 250 Hz, 6–7 dB in the frequencies of 500 and 1000 Hz and, almost negligible after the first cut-off frequency. Overall, The acoustic performance of the simple expansion silencer was poor such that the total insertion loss was about 4 dB.

**Fig. 15:**
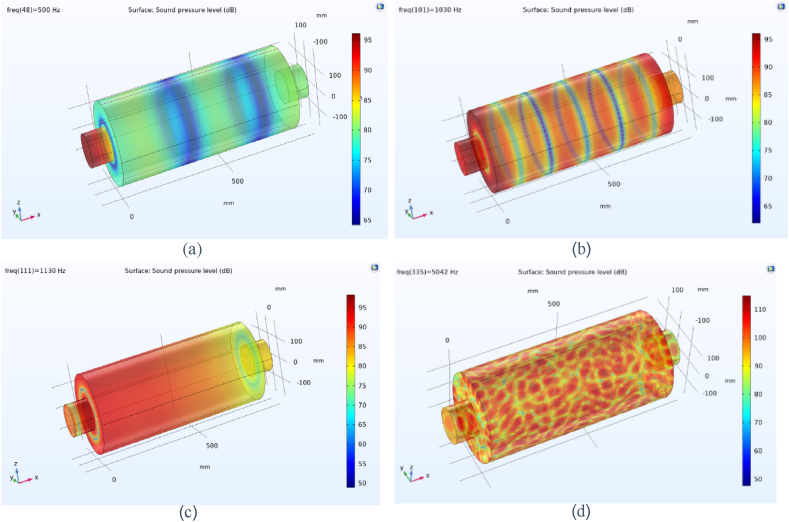
Sound pressure level distribution at various frequencies in a simple expansion silencer, a: 500 Hz, b: 1030 Hz, c: 1130 Hz, d: 5042 Hz.

The propagation of sound waves inside the simple expansion silencer up to the first cut-off frequency is in the form of flat and one-dimensional waves. As it can be seen in Fig. 15, domes, troughs and transverse waves have been formed along the simple expansion silencer at the frequency of 500 Hz. It is fundamental to note that as the frequency increases and approaches the frequency of 1030 Hz, the number of domes and troughs and transverse waves increase. At the first cut-off frequency, where the wavelength of the sound waves is equal to the diameter of the expansion silencer, dome and trough pattern disappears, sound pressure distribute widely along the silencer and transverse waves convert to longitudinal waves. This issue is clearly evident in the sound transmission loss curve of simple expansion silencer in Fig. 13. At higher frequencies (5042 Hz), longitudinal and transverse waves propagate in different directions inside the silencer, Fig. 15 d. So, with a few exceptions noise reduction performance of the simple expansion silencer at frequencies higher than the cut-off frequency was interestingly poor. As if the length of the silencer play as the main role in frequencies with the highest (blue points) and the lowest (red points) noise reduction, it would be possible to change the silencer length for noise reduction in a desired frequency. Another point for consideration is that the ratio of expansion silencer diameter to the inlet tube diameter is a crucial parameter to determine the range of frequencies that silencer performs well. With increasing the diameter of the expansion chamber to achieve more noise reduction, the first cut-off frequency moves toward low frequencies and the frequency range of silencer performance is reduced. Therefore, utilizing this kind of silencer can be limited to single frequencies depending on the length of the silencer.

**Fig. 16:**
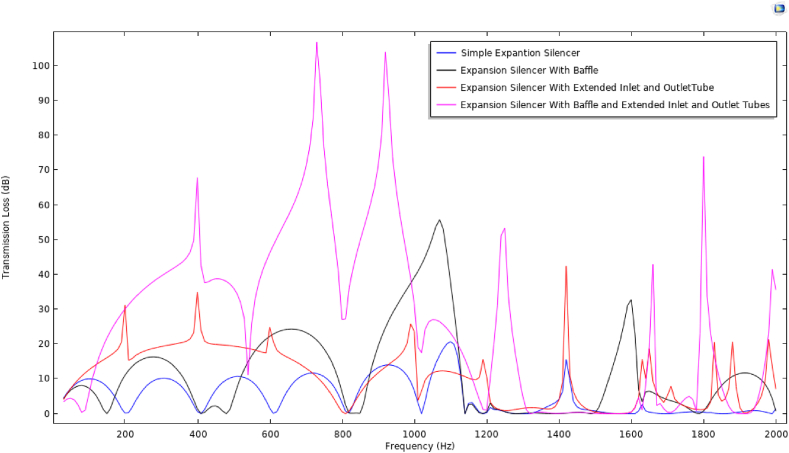
Modeled sound transmission loss curve of silencers with different configurations.

**Fig. 17:**
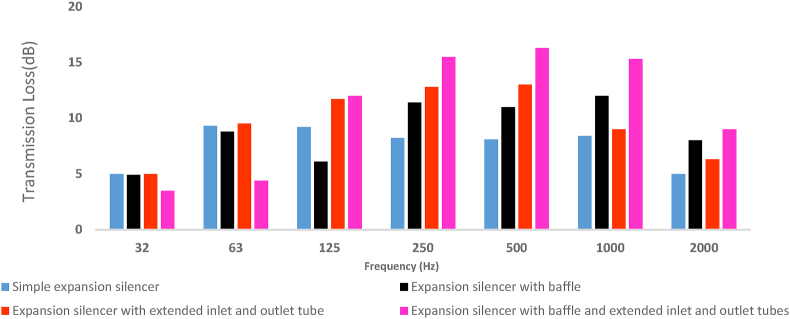
Modeled sound transmission loss of silencers with different configurations in octave frequencies.

Considering the effect of reactive elements at frequencies lower than the first cut-off frequency and as well as that the accuracy and precision of the modeling results up to the first cut-off frequency, to investigate carefully the effect of these elements and compare the performance of silencers, transmission loss curve and the sound transmission loss bar charts of the under studying silencers are illustrated in frequencies lower than 2000 Hz in Fig. 16, Fig. 17, respectively.

In comparison to simple expansion silencer, the baffled silencer transmission loss curve, as shown in Fig. 16, represents a decrease in the number of troughs and an increase in the height of the peaks in the frequencies before and after the frequency of 500 Hz. Moreover, fifty percent of the periodic troughs was removed and the periodic sound reduction pattern was changed in simple expansion silencer.

It is interesting to note that changing the length of the expansion chamber by adding a baffle in its middle caused the poor performance of the silencer at the frequency of 500 Hz and its adjacent frequencies. For instance, the transmission loss curve increased in the other investigated frequency (660 Hz) in this silencer. The function of the baffle as a noise barrier inside the expansion chamber increased the transmission loss curve in the medium frequencies. In contrast, baffle reduced the transmission loss curve at low frequencies (below 250 Hz) compared to a simple expansion silencer. In other words, adding a baffle to a simple expansion silencer increased the amount of transmission loss 11–12 dB in octave frequencies (250–1000) and caused a decrease in the transmission loss at 63 and 125 Hz frequencies compared to the simple expansion silencer (Fig. 17).

**Fig. 18:**
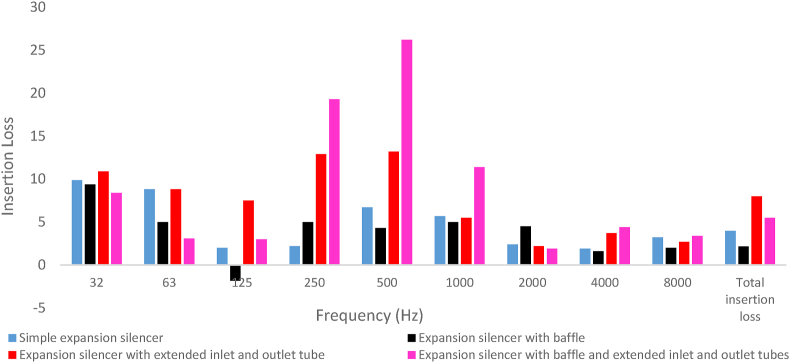
The measured insertion loss of the silencers with different configurations in octave frequencies.

Fig. 18 revealed the results of noise insertion loss charts as a function of frequency for various configuration of under studying silencers. From Fig. 18, the baffled silencer compared to the simple expansion silencer showed a decrease and an increase in noise insertion loss at frequencies 32–125 and 250 Hz, respectively.

**Fig. 19:**
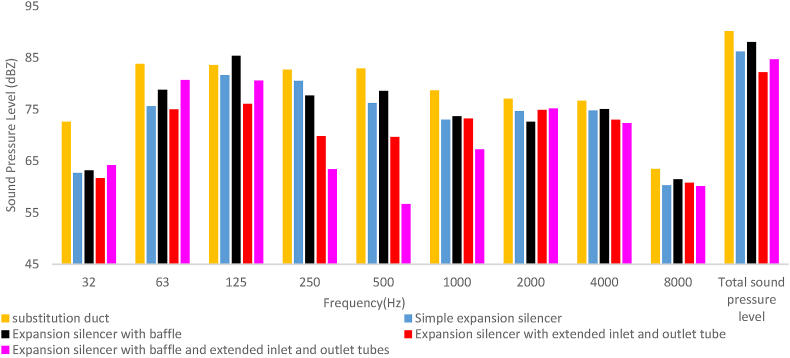
The measured sound pressure level of silencers with different configurations.

Experimental results demonstrated that the baffled silencer is the one with the lowest acoustic performance. The total sound pressure level before installing the silencer (with a substitution duct) and after installing the baffled silencer was measured to be 90.1 and 88 dB, respectively (Fig. 19). In other words, the insertion loss of baffled silencer was measured to be 2.1 dB. Although, the addition of the baffle resulted in increasing predicted transmission loss at frequency of 500–1000 Hz, the values of insertion loss in this frequency range decreased by 1–2 dB compared to the simple silencer. At the frequency of 125 Hz, this silencer not only did not reduce the noise, but also increased the sound pressure level by 3.8 dB. This poor performance reduced the overall performance of the silencer compared to the simple silencer.

Therefore, according to the obtained results, it can be concluded that adding a baffle inside the chamber and its location should be based on the target frequency and the length of the chamber. In this study, the baffle had not been designed for a specific target frequency, and the aim was only to investigate its effect on the performance of the simple silencer. So, adding a baffle in the middle part of the chamber accidently improved the performance of the simple expansion silencer in some frequencies and weakened it in others. Adding a baffle with a central hole in the middle part of the silencer operates like a sound barrier in the propagation path of sound waves inside the chamber and returns back of sound waves toward the sound source, similar to what happens at the end of a simple expansion chamber. This phenomenon leads to an increment in sound interference and eventually sound waves dissipate in some frequencies.

**Fig. 20:**
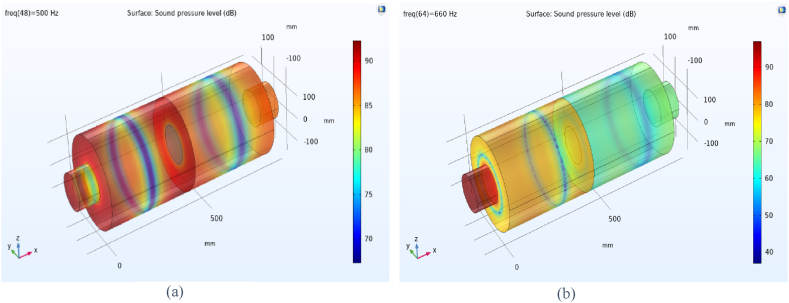
Distribution of sound pressure level in some frequencies in baffle expansion silencer, a: 500 Hz, b: 660 Hz.

The distribution of sound pressure level for baffled silencer at frequency 500 Hs was depicted in Fig. 20 a. It is essential to be noted that the addition of the baffle changes the length of the silencer and following that changes the target frequencies for noise reduction in the expansion silencer, so that the high sound pressure level at the output of this silencer has been observed. It indicates the escape of sound waves at frequencies of 500 Hz and of course the reduction of silencer performance.

The acoustic performance assessment of the baffled silencer at other frequencies such as 660 Hz (Fig. 20b) showed a decrease in the sound pressure level at the output of the silencer, which means better acoustic performance. The aforementioned results showed that the length of the silencer is an effective parameter on the target frequencies for noise reduction, therefore, installing the baffle at different locations along the expansion silencer length can lead to noise reduction at different frequencies through changing the length of the chamber and serializing the chambers. In Roy's study, adding a series or adding a central baffle decreased the transmission loss in low frequencies and increased the transmission loss in medium frequencies, which is in consistent with the results of our study [40].

It is fundamental to note that the addition of extended tubes to the simple expansion silencer resulted in the best acoustic performance at frequencies 32–1000 Hz and especially at frequencies 125–500 Hz. In Fig. 16, adding an extended tube at the inlet and outlet of the simple expansion silencer increased the transmission loss curve in low and medium frequencies. In addition, approximately 75% of the periodic troughs of the transmission loss curve compared to the simple expansion silencer was removed and a broadband sound transmission loss curve along the low and medium frequency range and up to the first cut-off frequency was predicted. The predicted sound transmission loss of extended tubes silencer in the octave frequencies, as illustrated in Fig. 17, showed a high rise in transmission loss in the frequency range of 32–1000 Hz compared to the simple silencer.

The extended tubes silencer compared to the simple expansion silencer demonstrated a tremendous increase in noise insertion loss in low and medium frequencies, Fig. 18. In comparison to simple expansion silencer, extended tubes showed more noise insertion of 5.5, 10.7 and 6.5 dB in the frequency 125, 250 and 500 Hz, respectively. As expected, extended tubes silencer total noise insertion loss increased by 4 dB. In fact, the length of extended tubes is principally calculated as a multiple of the length of the chamber, therefore, the length of the extended tubes plays an important role in optimizing the performance of the extended tubes silencer at the desired frequencies. Adding an extended tube which is equal to the one-half and one-quarter of the length of the chamber at the entrance and exit of the simple expansion silencer, respectively, acts as a resonator and improves the interference of sound waves and mitigates the energy of sound waves.

**Fig. 21:**
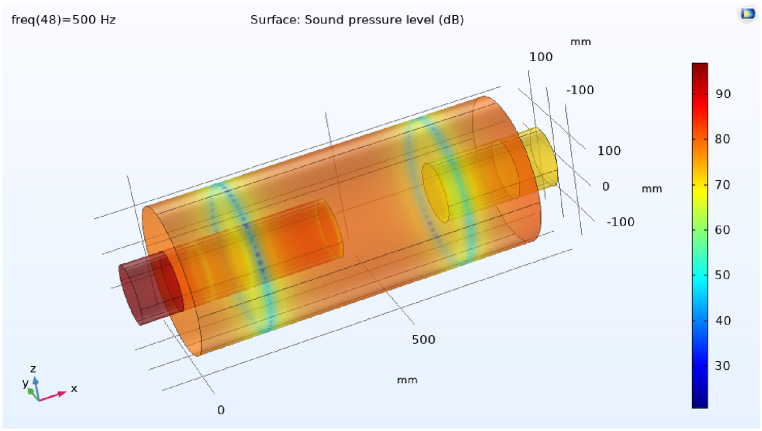
The distribution of sound pressure levels at a frequency of 500 Hz in an expansion silencer with extended inlet and outlet tubes.

If the length of chamber is considered to be fix and the entry and exit locations of the sound waves are changed, a wide frequency range of sound waves would be trapped between the tubes and the wall of the chamber. As illustrated in Fig. 21, the sound pressure level at the inlet and outlet of the silencer indicates the optimal performance at the frequency of 500 Hz. So, it can be seen that these lengths of the extended tubes at the inlet and outlet are suitable for noise reduction.

A good correlation and consistency were observed between the results of modeling and field measurements of simple expansion and extended tubes silencers. Gupta conducted a research on the simple expansion silencer, the expansion silencer with extended tube at the inlet, the expansion silencer with extended tube at the outlet, and the expansion silencer with extended tubes at both the inlet and outlet using the COMSOL software and experimentally. The expansion silencer with extended tubes at the inlet and outlet simultaneously had the highest noise reduction among the investigated silencers [14]. In Gupta's study, the length of the extended tubes at the inlet and outlet was taken similar to each other as one-fourth of the length of the expansion silencer length. However, in the present study, the length of the extended tubes at the inlet was half and at the outlet was one-fourth of the expansion silencer length. Comparing the results obtained from the transmission loss curve, we observed that the performance of the extended tubes silencer in the present study is significantly better than Gupta's silencer in terms of sound reduction in low and medium frequencies. Therefore, based on previous studies and the results of the present study, the length of the extended tubes and their desirable performance in low and medium frequencies are highly important and provide a solution for enhancing the performance of the silencer. It is interesting to note that different size of extended tubes is a critical factor in improving silencer performance especially in low and medium frequencies.

Adding two reactive elements at the same time, a baffle and extended tubes, to a simple expansion silencer results in the elimination of all periodic resonances and an increase in the transmission loss curve at medium-range frequencies up to the first cut-off frequency (Fig. 16). Examining the sound transmission loss at octave frequencies (Fig. 17) represented, a significant increase in the sound transmission loss of this silencer compared to the simple expansion silencer and other silencers. The presence of extended tubes in this silencer, which have relatively good performance at medium-range frequencies, and as well as the effect of the baffle at 250 Hz based on the length of the chamber and the combined effect of these elements, significantly increased the transmission loss at medium-range frequencies compared to the simple silencer.

Silencer with extended tubes and baffle experienced more noise insertion loss of 17, 19 and 5.7 dB at frequencies of 500 Hz, 250 Hz, and 1000 Hz, respectively, compared to the simple expansion silencer, as illustrated in Fig. 18.

The presence of the baffle in this silencer also had a negative impact on lower frequencies, resulting in a decrease in transmission loss at frequencies of 32 Hz, 63 Hz, and 125 Hz. Similar results have been obtained for insertion loss at these frequencies. Although, the overall performance (insertion loss) of this silencer improved by 1.5 dB compared to expansion silencer, the negative effect of the baffle on lower frequencies reduced the overall performance of this silencer by 2.5 dB compared to the silencer with extended tubes without a baffle. So, this silencer can be the best choice for a sound source with dominant frequencies in medium-range. In a study conducted by Middleburg to examine the acoustic performance of various types of simple expansion silencers, baffled silencers, and those with extended inlet and outlet tubes using CFD methods, it was found that baffles and extended tubes had similar effects on the performance of the silencer under investigation [11]. The combination of a baffle and extended tubes, and as well as that the functional mechanisms of these elements such as series arrangement, changes in chamber length, and new lengths of extended inlet and outlet tubes, increased the interference of sound waves, leading to a reduction in energy and sound pressure level compared to previous silencers.

**Fig. 22:**
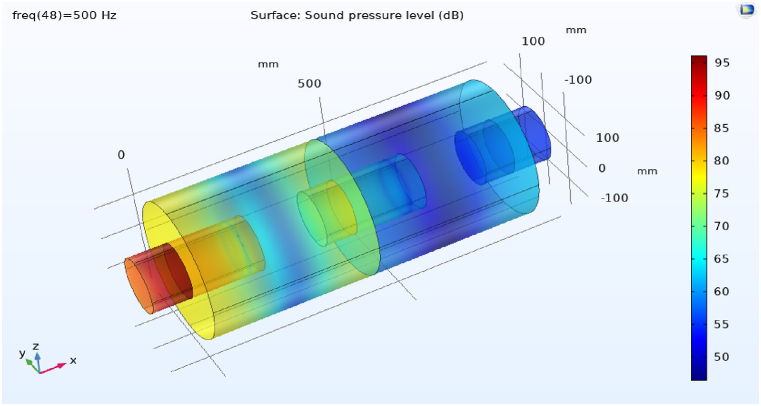
The distribution of sound pressure level at a frequency of 500 Hz in an expansion silencer with extended tubes and baffle.

The excellent performance of this silencer at 500 Hz is reported in Fig. 22, which can be compared by examining the sound pressure level at the outlet of the silencer with other silencers. The reduction of the entry and exit distances of sound waves into the chamber with the new lengths of extended tubes and the addition of baffles and changes in chamber length have resulted in a decrease in energy and sound pressure level at low and medium-range frequencies. Due to the long wavelengths of low and medium-range frequencies and assuming a fixed chamber diameter, adding extended tubes and baffles at the same time increases the interaction and breakdown of sound waves, ultimately reducing energy and sound pressure level at the silencer's outlet, thereby enhancing its acoustic performance.

**Fig. 23:**
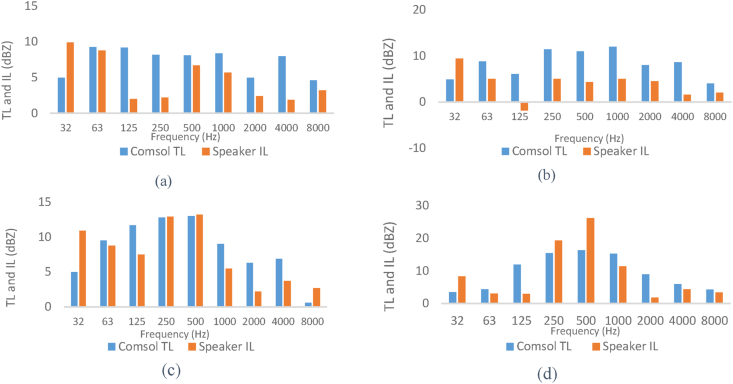
The results of insertion loss and modeled transmission loss of the silencer (a: simple expansion silencer, b: expansion silencer with baffle, c: expansion silencer with extended inlet and outlet tube, d: expansion silencer with baffle and extended inlet and outlet tubes).

Comparison of the results of modeled sound transmission loss and measured insertion loss of silencers in octave band frequencies is reported in Fig. 23. The results of insertion loss and transmission loss in the expansion silencer with extended tubes are in close octave frequencies and the results are consistent. For example, at the frequency of 250 Hz, the results of transmission loss and insertion loss are 12.8 and 12.9, respectively, and at the frequency of 500 Hz, the values of transmission loss and insertion loss are 13 and 13.2 dB, respectively. In the expansion silencer with baffle and extended tubes, the difference in the values of insertion loss and transmission loss at frequencies of 250, 500, 1000 Hz is small and they almost match each other.

Comparing the insertion loss and sound transmission loss of a simple expansion silencer and a baffle-equipped silencer in octave band frequencies shows that adding a baffle to the simple expansion silencer reduces the sound transmission loss at frequencies of 32, 63, and 125 Hz. At a frequency of 125 Hz, there is a significant difference in the results of insertion loss and sound transmission loss between the simple expansion silencer and the baffle-equipped silencer.

The measurement of the acoustic performance of silencers in this article is conducted in the frequency range of 32–8000 Hz, and the performance of the silencer even partially affects the overall results at frequencies higher than the first cut-off frequency. However, the model results are only applicable and comparable up to the first cut-off frequency due to the use of one-dimensional flat waves in the modeling, in reality as the frequency increases it probably increases scattering non-flat sound waves, which can significantly affect the insertion loss (IL). Nevertheless, there is a correlation and regular pattern in the increase and decrease of the silencer performance in IL and transmission loss (TL) graphs in the corresponding frequencies, as shown in Fig. 23. For example, in the expansion silencer with extended tubes, good agreement and correlation were observed between the modeling results and field experiments. Previous studies on the acoustic performance of silencers have also reported differences in IL and TL values at certain frequencies, especially at 125 Hz [41].

### 3.3 Pressure loss performance

**Fig. 24:**
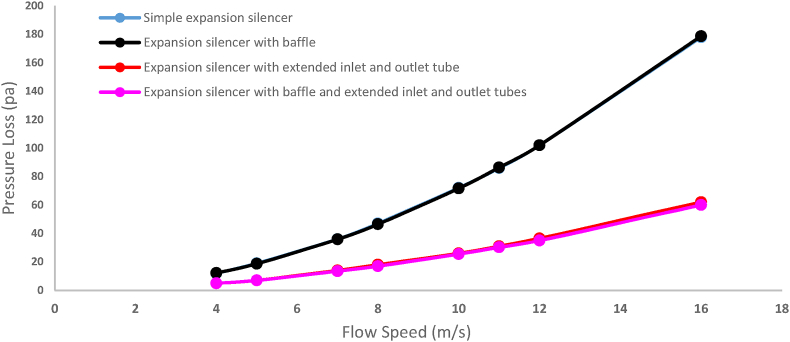
The results of modeling pressure loss in silencers with various configurations.

The results of pressure loss modeling for the studied silencers are reported in Fig. 24. The lowest and highest pressure loss at various flow velocities observed for the baffled silencer with extended tubes and the expansion baffle silencer, respectively. The pressure loss values for the baffled silencer with extended tubes at flow speeds of 4, 8, 11, and 16 were 5, 17, 30.3, and 60 Pa, respectively. The corresponding pressure loss values for the expansion baffle silencer were 12.2, 46.5, 86.3, and 187.7 Pa at the same flow speeds.

**Fig. 25:**
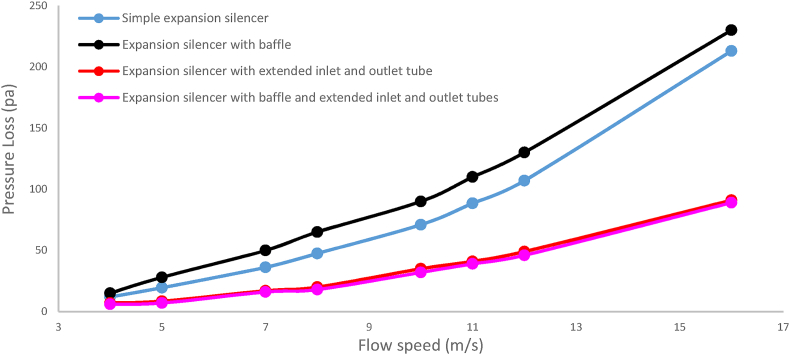
The results of measured pressure loss in silencers with various configurations.

Furthermore, the experimental measurement results of the pressure loss for the silencers are reported in Fig. 25. The lowest and highest pressure loss at the measured flow speeds were observed, respectively, for the expansion baffle silencer with extended tubes and the expansion baffle silencer. The pressure loss values for the baffled silencer with extended tubes at flow speeds of 4, 8, 11, and 16 were measured to be 6, 18, 39, and 89 Pa, while for the expansion baffle silencer, the corresponding values were 15, 65, 110, and 230 Pa at the same flow speeds. In conclusion, the obtained results of the silencer pressure loss show a correlation between the modeling and measurement results.

**Fig. 26:**
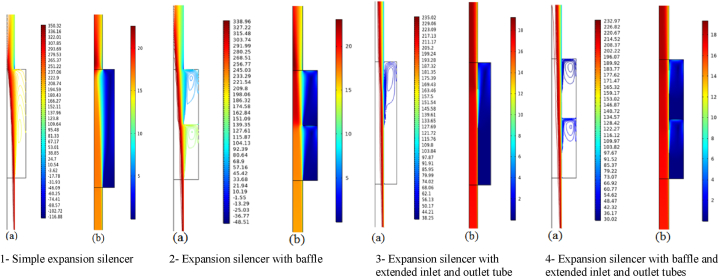
The instantaneous distribution of air flow speed (a) and total pressure (b) inside silencers.

Adding extended tubes to a simple expansion silencer has been resulted in a decrease in the speed and overall pressure loss of the airflow inside the silencers. The investigation of pressure loss in the simple expansion silencer showed that an increase in airflow speed at the end section and the constricted section of the expansion chamber led to an increase in pressure loss. Adding baffles, considering the increased number of these constrictions, and with the central hole diameter of the baffle equal to the inlet and outlet diameter of the silencer, caused a partial increase in airflow speed and further pressure loss. Experimental results also indicated that the pressure loss in the baffle silencer was slightly higher than that in the simple expansion silencer for all examined flow speeds. This issue could be mitigated by increasing the diameter of the central hole of the baffle. The pressure loss results in Fig. 24 for an airflow speed of 8 m per second at the inlet, of the simple expansion silencer and the extended tube silencer were 47 and 18 Pa, respectively, in the modeling section, and 47.5 and 20 Pa, respectively, in the experimental results section (Fig. 25). These results demonstrated that the significant improvement in pressure loss reduction achieved by the extended tubes. The pressure loss results of the silencer with extended input and output tubes showed that adding extended tubes to the simple expansion silencer resulted in a reduced pressure loss due to creating a flow condition similar to the movement of air within the tube, preventing the expansion and contraction of the flow as occurs in the simple expansion silencer. The instantaneous speed distribution of the airflow (a) and the overall pressure distribution (b) inside the silencers under considerations where the inlet airflow speed is 16 m per second, and the fan operates at maximum power, are shown in Fig. 26. It is evident from Fig. 26 that adding extended tubes leads to a decrease in airflow speed at the outlet and a reduction in pressure loss. For example, at an airflow speed of 8 m per second, the simulated and experimental pressure loss were obtained as 18 Pa for the baffle and extended tube expansion silencer. This silencer had the lowest pressure loss among all the examined silencers. Therefore, it can be concluded that adding extended tubes and baffles to the simple expansion silencer reduces the pressure loss due to the reduced distance between the openings of the extended tubes and the ease of airflow transfer between them, compared to all the studied silencers. This finding is consistent with the Midelberg study, where the use of extended tubes resulted in a reduction in pressure loss compared to the simple expansion silencer [11].

**Fig. 27:**
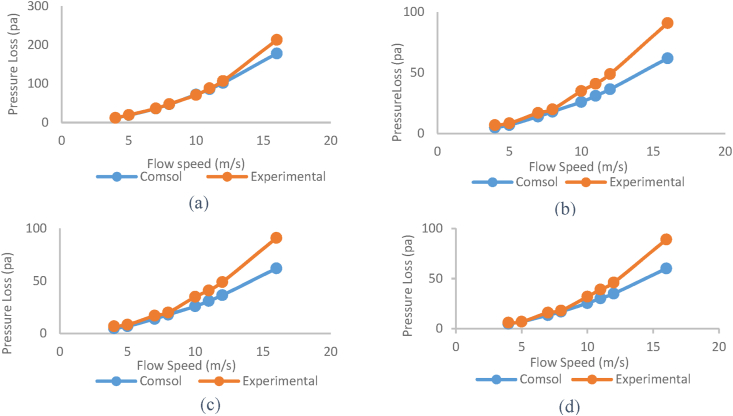
Modeled and experimental pressure loss of silencers (a: simple expansion silencer, b: expansion silencer with baffle, c: expansion silencer with extended inlet and outlet tube, d: expansion silencer with baffle and extended inlet and outlet tubes).

The modeled and experimental results of pressure loss in the silencers have been reported in Fig. 27. The modeled and experimental values showed some differences at low flow speeds, sometimes relatively minor. However, with an increase in air flow speed, the difference between the modeled and experimental pressure loss values increased.

By comparing the pressure loss of the silencers in Fig. 27, the results are similar in most flow speeds, particularly at low speeds and sometimes very close to each other. However, with increasing flow speed, due to factors such as tube irregularities, connections, etc., which increase flow turbulence, the experimental results deviate from the modeled values. To some extent, the analysis of the performance of the reactive elements on the pressure loss of the silencers indicates consistency between the modeling and experimental results.

In this study, there were limitations in providing requested conditions based on ISO 3744 and ISO 3746 standards for measuring insertion loss such as an environment without reflective surfaces, the influence of wind gusts, and transient sound sources like motorcycle and car noises. These limitations increased the number and duration of measurements. It is suggested to use empirical methods employing microphones inside the ducts to reduce measurement errors when measuring transmission loss. Furthermore, it is recommended to investigate the effects of increasing the baffled hole diameter compared to the inlet diameter and increasing the number of baffles in future studies.

## 4 Conclusion

The results of this paper show that the addition of an extended tube to the simple expansion silencer, by increasing the transmission and insertion loss at low and medium frequencies, increases the acoustic performance of the silencer in the frequency ranges up to the first cut-off frequency and also reduces the pressure loss compared to the simple expansion silencer. The addition of a baffle with a central cavity alone increases the transmission and insertion loss at individual frequencies compared to a simple expansion silencer, but reduces the acoustic performance of the entire silencer by reducing the transmission and insertion loss at low frequencies and also increases the pressure loss. The addition of baffle and extended tubes increase the transmission and insertion loss in the medium frequencies and decreases the pressure loss of the silencer compared to the simple expansion silencer and the other silencers studied. The presence of a baffle in this silencer and its negative effect by reducing the transmission and insertion loss at low frequencies reduces the acoustic performance of the silencer compared to the silencer with an extended tube. The results of modeling using COMSOL software as a finite element method have also shown that this method can save time and costs and avoid the errors and problems of the experimental test method.

## Funding statement

This study has been performed within the framework of a faculty research project under the code of ethics Committee of IR. QUMS.REC.1398.241 (18,243). It has been supported by the Research Vice-Chancellor of 10.13039/501100006396Qazvin University of Medical Sciences.

## Additional information

No additional information is available for this paper.

## Author contribution statement

Motaleb rahimi: Conceived and designed the experiments; Performed the experiments; Analyzed and interpreted the data; Contributed reagents, materials, analysis tools or data; Ali safary: Contributed reagents, materials, analysis tools or data. Saeid Ahmadi: Conceived and designed the experiments; Analyzed and interpreted the data; Wrote the paper.

## Data availability statement

The data that has been used is confidential.

## Declaration of competing interest

The authors declare that they have no known competing financial interests or personal relationships that could have appeared to influence the work reported in this paper.

## Nomenclature

TL =: Transmission Loss (dB)
IL =: Insertion Loss (dB)
K =: wave number
λ =: wavelength (m)
C =: speed of the sound (m/s)
F =: Frequency
ω =: angular frequency
F_up_ =: cut-off frequency
L_g,a_ =: length of extended inlet tube (m)
L_g,b_ =: length of extended out let tube (m)
L =: length of silencer expansion chamber (m)
δ =: final correction factor
D =: diameter of an expansion silencer (m)
t_w_ =: is the thickness of the inlet and outlet tube(m)
d =: diameter of the inlet and outlet tube (m)
p =: sound pressure (pa)
ρ =: material density (kg/m^3^)
P_in_ =: acoustic power in the inlet of silencer
P_out_ =: acoustic power in the outlet of silencer
u =: average velocity (m/s)
μ =: fluid's dynamic viscosity (kg/m.s)
μ_T_ =: Turbulent viscosity (kg/(m.s))
K =: Turbulent kinetic energy (m^2^/s^2^)
ε =: Turbulent dissipation rate (m^2^/s^3^)
Pk =: turbulence kinetic energy that describes the balance between the production, dissipation, and transport of turbulence kinetic energy in a fluid
Lw =: sound power level
$\bar{\text{pt}}$ =: mean total pressure
Δ p =: pressure loss (Pressure difference upstream and downstream)
